# Unworn cartilage in total knee arthroplasty strongly deviates from 2 mm: MRI, robotic‐assisted and ruler assessment

**DOI:** 10.1002/ksa.12671

**Published:** 2025-04-01

**Authors:** Antonio Klasan, Victoria Anelli‐Monti, Alexander Johannes Nedopil

**Affiliations:** ^1^ AUVA UKH Steiermark Graz Austria; ^2^ Johannes Kepler University Linz Linz Austria; ^3^ Adventist Health Memorial Hospital Lodi California USA; ^4^ University of Würzburg Würzburg Germany

**Keywords:** cartilage, kinematic alignment, MRI, robotic‐assisted surgery, total knee arthroplasty, wear

## Abstract

**Background:**

Kinematic alignment (KA) in total knee arthroplasty (TKA) aims to resurface the articular surface of the pre‐arthritic knee. One cornerstone of KA TKA and the basis for conventional instrumentation (CI) is a 2 mm cartilage thickness of the healthy distal femur. This study of image‐based (CT) robotic‐assisted surgery (RAS) TKA determined (1) the difference in measuring cartilage thickness using preoperative MRI, the RAS registration probe and MRI and (2) the percentage of knees with a <1.5 or >2.5 mm thickness of the unworn cartilage.

**Methods:**

Forty‐two patients undergoing a TKA using an image‐based RAS were included. Preoperative long‐leg radiographs provided the limb phenotype. The two zones of interest for cartilage thickness were the distal unworn (DU) and posterior unworn (PU) condyle. The preoperative MRI provided cartilage thickness values for both zones. Intraoperatively, the surgeon identified the zones without cartilage wear (healthy cartilage) and measured the distance between the cartilage and bone with the RAS registration probe. After resecting the condyles, a ruler was provided to measure the cartilage thickness reference.

**Results:**

Mean patient age was 68.2 ± 4.1 years, and gender was evenly distributed. According to the phenotype classification, there were 8 NEU_HKA_0°, 13 VAR_HKA_3°, 9 VAR_HKA_6°, 5 VAR_HKA_9°, 4 VAL_HKA_3°, 2 VAL_HKA_6° and 1 VAL_HKA_9° patient. The difference between measurement techniques was not statistically significant for both zones (DU *p* = 0.960, PU *p* = 0.267). In seven patients (16.7%), the DU cartilage thickness was >2.5 mm measured with any of the three techniques, and up to 50% of patients had unworn cartilage thickness <1.5 mm, depending on the zone and measurement technique.

**Conclusion:**

There is a significant variation of unworn cartilage thickness from the assumed 2 mm, with a significant proportion (up to 67.7%) having >2.5 or <1.5 mm. If KA is applied, additional preoperative or intraoperative measurements might provide valuable information to avoid the risk of femoral component malposition.

**Level of Evidence:**

Level II, diagnostic study.

**Abbreviations**
CI: conventional instrumentation
DL: distal lateral
KA: kinematically aligned
PL: posterior lateral
RAS: robotic assisted surgery
TKA: total knee arthroplasty

## INTRODUCTION

Arguably the most commonly used technique to deliver kinematically aligned (KA) total knee arthroplasty (TKA) is using conventional instruments with caliper verifications [9]. The principle of KA is to restore the native knee kinematics by replacing the removed bone and cartilage in the same thickness as the implant, thus safely restoring the native kinematics of the knee, irrespective of the native alignment [10, 18]. Since the thickness of the cartilage varies depending on the wear pattern, the cartilage on the worn side is removed. Based on multiple magnetic resonance imaging (MRI) cartilage studies, the thickness on the unworn side is assumed to be 2 mm [2, 3, 16, 22]. Thus, the difference between the worn and unworn side of the joint surface should theoretically be 2 mm, in case no bone loss is present.

The difference of 2 mm is the basic principle of modern conventional instrumentation (CI) for KA [6]. The use of image‐based robotic‐assisted surgery (RAS) systems revolves around bony referencing [19]. Any difference in cartilage thickness between the medial and lateral sides is automatically adjusted for [21]. A recent systematic review of 8170 MRIs demonstrated significant variation in cartilage thickness, recommending intraoperative assessment of cartilage thickness due to potential misalignment errors made by the assessment [5]. Currently, no TKA system takes the cartilage thickness into consideration. If true native kinematics is to be restored, the implant should replace the bone and the native cartilage.

The purpose of the present study was to investigate cartilage thickness in patients undergoing TKA using MRI, a robotic tool, and an intraoperative ruler.

We hypothesized that the unworn cartilage of the distal and posterior femoral condyles is not 2 mm thick in every patient, questioning the established assumption of a 2 mm difference between the worn and unworn side.

## PATIENTS AND METHODS

### Patients

This prospective single‐centre study was conducted between 12/2023 and 02/2025 under a prospective data collection ethics board approval (AUVA Ethics Board 17/2021). The centre has a prospective Ethics Board approval on 17/2021 for RAS TKA. In all cases, RAS‐TKA was performed (MAKO Rio, Stryker) using Triathlon (Stryker) implants. We recorded age and gender, and HKA according to the phenotype classification [8].

**Figure 1:**
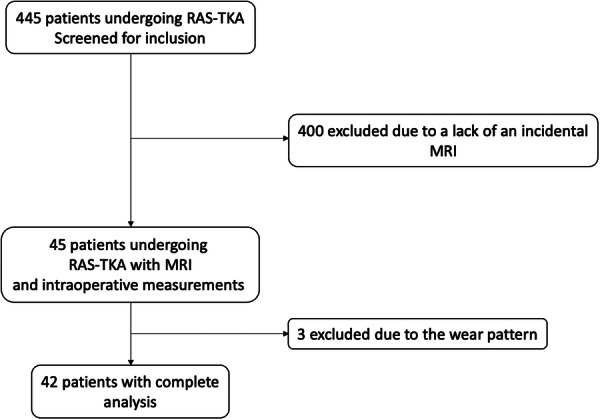
Flow chart of patient inclusion. MRI, magnetic resonance imaging; RAS, robotic‐assisted surgery; TKA, total knee arthroplasty.

Patients undergoing RAS‐TKA for primary osteoarthritis (OA) were screened for inclusion. Due to the purpose of the study, patients without incidental prior MRI of the affected knee [20] and patients with a generalized OA pattern without an unworn side were excluded. After applying the inclusion and exclusion criteria, 42 patients were analyzed (Figure 1). MRI is occasionally performed by the general practitioner prior to referral.

### Measurement technique

The two points of interest for cartilage thickness measurements were in the varus OA knee, the distal lateral (DL) and posterior lateral (PL) condyles, representing unworn surfaces, identified using the best‐fit circle method [10, 16].

The points were identified on preoperative 3 T T2‐weighted coronal and sagittal sequences. The sequence thickness was 1.5 mm (Siemens Somatom MRI, Siemens AG). Once the points were identified, the cartilage thickness for each point was measured by two assessors (A.K. and A.J.N.), with 2 weeks between assessments.

**Figure 2:**
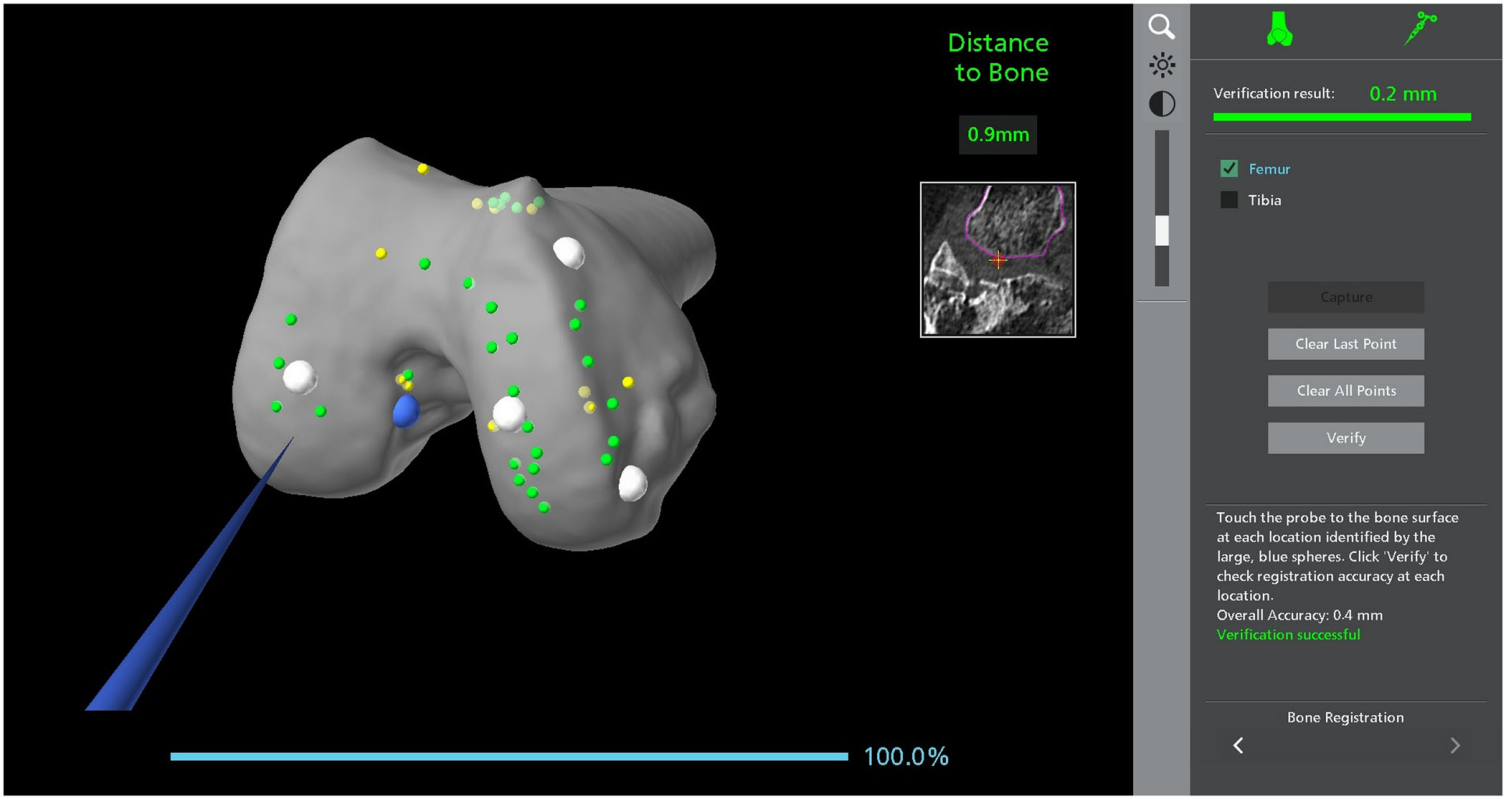
Cartilage thickness measurement using the robotic probe.

Intraoperative measurements were performed using the MAKO robotic software (Stryker) in an off‐label manner. More specifically, during the registration process, a blue sharp probe takes 40 points per bone, directly on bone, piercing through cartilage in the non‐worn areas [19]. This registration is verified by checking the distance to bone on an additional 5 points. During this part, the system directly shows the live distance to the bone. The probe was utilized to measure the distance to the bone on the 2 points of interest by being held directly at the cartilage level without piercing through bone (Figure 2). The measurements were repeated two times for all cases by a single surgeon (A.K.).

**Figure 3:**
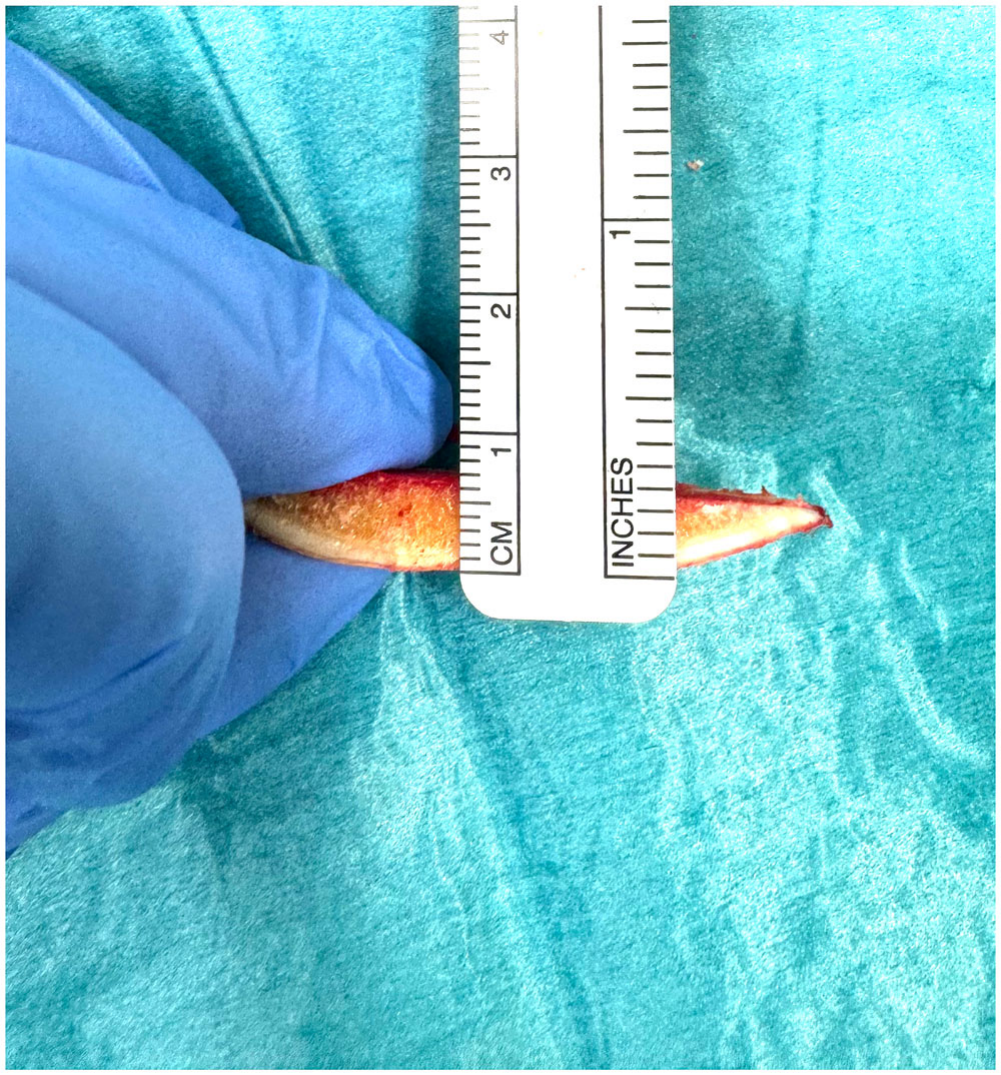
Caliper measurement of cartilage thickness.

Once the cuts were performed, they were halved using an oscillating saw at each point of interest and measured using a ruler to calibrate the photo‐documented cartilage measurement (Figure 3). The measurements were repeated two times for all cases by two surgeons (A.K. and A.J.N.). The measurement resolution of calipered measurement was 0.5 mm, and MRI and robotic techniques were rounded to 0.1 mm.

## STATISTICAL ANALYSIS

Inter‐ and intraobserver agreement was evaluated using the intraclass correlation coefficient (ICC). The data are presented as mean (±standard deviation). For each zone, the differences in cartilage thickness between each measurement were analyzed using repeated measurements analysis of variance with Bonferroni correction for multiple measurements. For the violated sphericity assumption, the Greenhouse‐Geiser estimate was used [1]. To account for the potential intraoperative error using conventional instruments, the percentage of cases with unworn thickness >2.5 and <1.5 mm was reported. To test the hypothesis of a uniform 2 mm worn–unworn difference, an a priori power analysis as an equivalence study with a continuous outcome was performed. Using an *α* of 0.05 and a *β* of 0.9, a standard deviation of 0.4 mm, and an equivalence limit of 0.5 mm, 28 patients were needed to demonstrate a significant difference. Statistical analysis was performed using SPSS 29 (IBM). Statistical significance was set at *p* < 0.05.

## RESULTS

### Patients

The mean patient age was 68.2 ± 4.1 years; gender was evenly distributed, 21 female and 21 male patients. In terms of HKA, there were 8 NEU_HKA_0°, 13 VAR_HKA_3°, 9 VAR_HKA_6°, 5 VAR_HKA_9°, 4 VAL_HKA_3°, 2 VAL_HKA_6° and 1 VAL_HKA_9° patient.

### Differences between measurement techniques

There were differences for each zone of interest in all techniques (Table 1). The differences were statistically significant but of low clinical relevance.

**Table 1:** Repeated measurements ANOVA analysis of the zones of interest.

Zone of interest	Distal unworn	Posterior unworn
Differences between groups	*p* < 0.001	*p* < 0.001
MRI measurement values (mm)	1.83 ± 0.47	1.65 ± 0.42
Robotic probe measurement values (mm)	1.90 ± 0.47	1.72 ± 0.39
Ruler measurement values (mm)	1.73 ± 0.50	1.57 ± 0.43
Effect size (F)	21.017	17.690

Abbreviations: ANOVA, analysis of variance; MRI, magnetic resonance imaging.

#### Cartilage thickness

Distally, 16.7% of patients had >2.5 mm unworn cartilage thickness, compared to 0% posteriorly, regardless of the measurement technique (Table 2). Conversely, up to 35% of patients had unworn cartilage thickness <1.5 mm distally and up to 50% posteriorly, depending on the measurement technique (Table 2).

**Table 2:** Percentage of cases with >2.5 and <1.5 mm unworn cartilage thickness.

	>2.5 mm			<1.5 mm		
	MRI	Robotic probe	Ruler	MRI	Robotic probe	Ruler
Distal unworn	7/42 (16.7%)	7/42 (16.7%)	7/42 (16.7%)	15/42 (35%)	9/42 (21.5%)	7/42 (16.7%)
Posterior unworn	0/20 (0%)	0/20 (0%)	0/20 (0%)	21/42 (50%)	12/42 (28.5%)	12/42 (28.5%)

Abbreviation: MRI, magnetic resonance imaging.

### Consistency of measurements

The intra‐ and interobserver ICCs for the MRI measurements were 0.97 and 0.93, for the ruler measurements 0.95 and 0.85, respectively, and the intraobserver ICC for the probe measurements was 0.93.

## DISCUSSION

The most important finding of the present study is the significant deviation of cartilage thickness of the unworn side in patients undergoing TKA, from the assumed uniform 2 mm. Distally, the cartilage thickness was thicker than 2.5 mm in 15% of patients and thinner than 1.5 mm in 24%–40%. Posteriorly, no patient had a thickness of more than 2.5 mm of cartilage thickness, but up to 50% of patients had a thickness of thinner than 1.5 mm of cartilage thickness. The difference in thickness measured by the three techniques was statistically significant but arguably clinically irrelevant.

KA is most commonly performed using conventional instruments [9]. To facilitate the process and the decision‐making, a distal and posterior resection block is mounted flush with the distal and posterior joint line, respectively. Instead of determining the resection angle to the anatomical‐mechanical axis and the posterior condylar axis, the surgeons can orient themselves on the wear pattern. The resection block has the options of unworn–unworn, worn–worn, and, most commonly, unworn‐worn. The difference in resection thickness between worn and unworn is typically 2 mm [9]. The surgeon then orients the distal and posterior resection according to the wear pattern, thus theoretically replacing what was removed and worn out. The resection thickness is then verified using a calliper [9]. The present study demonstrates that cartilage thickness can be 2.5 mm or more on the unworn side and less than 1.5 mm. Assuming the medially worn distal and posterior cartilage in a varus OA knee has been removed to expose subchondral bone and assuming the cartilage thickness of the medial condyle equalled the cartilage thickness of the lateral condyle, a 1 mm cartilage thickness of the healthy lateral condyle would put the femoral component into more valgus and more internal rotation, thus altering the kinematics.

A theoretical advantage of image‐based systems is the reference from the bone and, thus, more precision [25]. A prerequisite is correct alignment and balancing target [7]. As demonstrated by the present study, the unworn cartilage thickness can vary. The resection should therefore be adjusted to accommodate cartilage thickness, which is not the case in any of the RAS‐TKA alignment strategies [12, 14, 21]. The typical starting point is an assumption of 1 or 2 mm cartilage thickness uniformly on all zones of interest [12, 14, 21]. Depending on the true cartilage thickness and no bone loss, the joint line can be proximalized or distalized, which alters the kinematics of the knee [10, 11] and can influence results [13].

Using CI, the cartilage thickness of the posterior condyles has been demonstrated to significantly influence femoral component rotation, thus altering kinematics [15, 17, 23]. Nam et al. demonstrated that cartilage and bone wear are predictable, presumably comparing the medial and lateral sides [16]. More than 90% of patients had <1 mm of wear on the medial side for varus knees and between 55% and 78% on the lateral side for valgus knees. The authors recommend compensating for cartilage wear only, when performing KA, which they measured to be on average 2 mm [16]. The caveat in that study is the use of MRI.

The theoretical advantage of image‐based robotics is the bony referenced‐based balancing and subsequent bony cuts [19]. The surgeon should theoretically check the unworn thickness first, compare it with the worn thickness in all ‘unworn’ zones of interest and adjust the starting position accordingly, under the assumption the cartilage thickness used to be uniform. No current robotic system has a workflow that accommodates cartilage thickness [4].

Several limitations need to be noted. The number of patients is low; however, due to strict inclusion and exclusion criteria and the power analysis performed, the study demonstrates the variability adequately. Clinical data were not collected, and the clinical implications remain unclear. Since functional alignment was performed in all cases, the intraoperative position is intraoperatively adjusted to match the soft tissue envelope almost uniformly [24], thus altering the position away from the KA position. The true clinical implication would be to compare KA using a standard 2 mm worn–unworn difference with KA using a measured worn–unworn difference. Only 10% of knees had a preoperative MRI, indicating the possibility of selection bias; however, other large MRI studies have demonstrated very similar results [5]. Bone loss, another parameter that is impossible to measure and needs to be assumed, has not been observed nor addressed in the present study. The RAS system used in the study is not primarily designed for this purpose. However, it tolerates a 0.1 mm error, henceforth the measurements can be used intraoperatively, off‐label, to aid in the decision of the implant starting position.

## CONCLUSION

If a KA strategy is applied, the assumption of a 2 mm worn–unworn difference might lead to a different implant alignment than planned. Additional preoperative or intraoperative analysis and a modification of the instrumentation to create a 1 and 3 mm difference is recommended.

## AUTHOR CONTRIBUTIONS

Antonio Klasan and Alexander Johannes Nedopil conceived the study. All three authors took the measurements, and Victoria Anelli‐Monti and Antonio Klasan conducted the analysis. Antonio Klasan wrote the first draft, and the coauthors revised it. All authors have read and approved the manuscript.

## CONFLICT OF INTEREST STATEMENT

Antonio Klasan is an associate editor for the *Journal of Knee Surgery* and an Editorial Board Member of *Archives of Orthopaedic and Trauma Surgery* and *Knee Surgery, Sports Traumatology, Arthroscopy*. He has been paid to present by Arthrex and Implantcast. Alexander Johannes Nedopil is a Medacta, Think Surgical, Microport, Smith and Nephew consultant. The remaining author declares no conflicts of interest.

## ETHICS STATEMENT

The study was performed under the approval of a prospective data collection ethics board (AUVA Ethics Board 17/2021). All patients provided written consent to participate.

## Data Availability

Data are available upon request.
